# Snake Venom Proteomics, Immunoreactivity and Toxicity Neutralization Studies for the Asiatic Mountain Pit Vipers, *Ovophis convictus*, *Ovophis tonkinensis*, and Hime Habu, *Ovophis okinavensis*

**DOI:** 10.3390/toxins13080514

**Published:** 2021-07-23

**Authors:** Choo Hock Tan, Praneetha Palasuberniam, Kae Yi Tan

**Affiliations:** 1 Venom Research, Toxicology Research Lab, Department of Pharmacology, Faculty of Medicine, University of Malaya, Kuala Lumpur 50603, Malaysia; praneetha@ums.edu.my; 2 Department of Biomedical Sciences, Faculty of Medicine and Health Sciences, University Malaysia Sabah, Kota Kinabalu 88400, Malaysia; 3 Protein and Interactomics Lab, Department of Molecular Medicine, Faculty of Medicine, University of Malaya, Kuala Lumpur 50603, Malaysia

**Keywords:** snakebite envenomation, venomics, procoagulant, antivenom, mountain pit viper

## Abstract

Snakebite envenomation is a serious neglected tropical disease, and its management is often complicated by the diversity of snake venoms. In Asia, pit vipers of the *Ovophis* species complex are medically important venomous snakes whose venom properties have not been investigated in depth. This study characterized the venom proteomes of *Ovophis convictus* (West Malaysia), *Ovophis tonkinensis* (northern Vietnam, southern China), and *Ovophis okinavensis* (Okinawa, Japan) by applying liquid chromatography-tandem mass spectrometry, which detected a high abundance of snake venom serine proteases (SVSP, constituting 40–60% of total venom proteins), followed by phospholipases A_2_, snake venom metalloproteinases of mainly P-III class, L-amino acid oxidases, and toxins from other protein families which were less abundant. The venoms exhibited different procoagulant activities in human plasma, with potency decreasing from *O. tonkinensis* > *O. okinavensis* > *O. convictus*. The procoagulant nature of venom confirms that consumptive coagulopathy underlies the pathophysiology of *Ovophis* pit viper envenomation. The hetero-specific antivenoms *Gloydius brevicaudus* monovalent antivenom (GbMAV) and *Trimeresurus albolabris* monovalent antivenom (TaMAV) were immunoreactive toward the venoms, and cross-neutralized their procoagulant activities, albeit at variably limited efficacy. In the absence of species-specific antivenom, these hetero-specific antivenoms may be useful in treating coagulotoxic envenomation caused by the different snakes in their respective regions.

## 1. Introduction

Snakebite envenomation affects poor and marginalized populations in the tropics and subtropics most heavily, killing more than 100,000 people annually, and with three times as many suffer chronic complications [1,2,3,4]. In 2017, the World Health Organization formally recognized snakebite envenomation as one of the most neglected tropical diseases and called for a global strategy to overcome the problem [5]. This global health crisis remains to be adequately addressed with a comprehensive solution.

The management of snakebite envenomation tends to be complicated by the great diversity and variable distribution of venomous snakes in different regions. Knowledge gaps in the identity, geographical distribution, and venom properties of specific species limit our understanding of the pathophysiology of envenomation, and this consequently hinders appropriate treatment [3]. Studies have extensively shown that venom composition between closely related species, or even within a single species from different populations, can vary considerably due to their adaptation to different ecological niches [6,7,8]. Snake venom variation has deep implications for the utility of antivenom against snakes (either of the same species or different populations) whose venoms are not included in the immunization process during antivenom production. Moreover, the supply and species coverage of antivenom products in most regions are inadequate, as antivenoms are produced by only a handful of domestic manufacturers in a few countries, with products indicated for selected important species in their respective countries [9].

**Figure 1:**
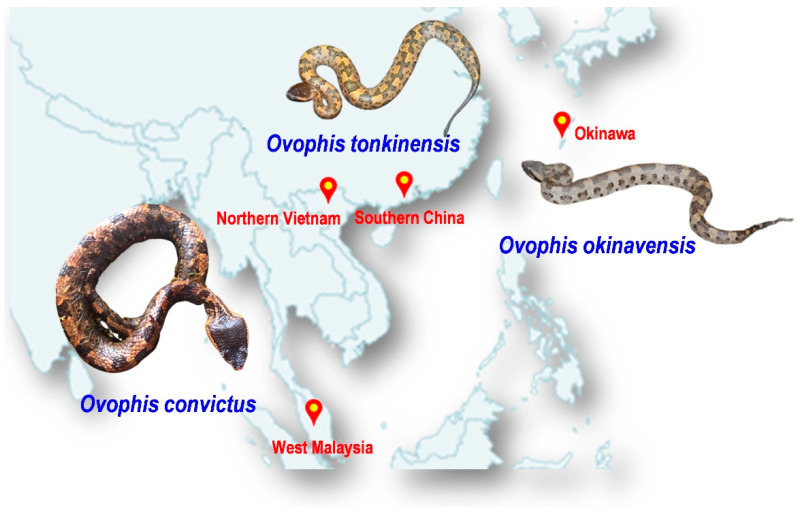
Illustration of three *Ovophis* pit viper species studied in the current work: *Ovophis convictus* from West Malaysia, *Ovophis tonkinensis* from northern Vietnam and southern China, and *Ovophis okinavensis* from Okinawa, Japan. Photo credits: Choo Hock Tan (*O. convictus*), Abdel Bizid (*O. tonkinensis*), Wikimedia (*O. okinavensis*).

The abovementioned issues undermine the effort to improve the management of snakebite envenomation in prevalent areas, including Southeast Asia and East Asia, where agricultural activities are intensive, venomous snakes are abundant and diverse, yet suitable antivenom products are limited [10]. One of the prime examples of medically important yet lesser-known venomous snakes in the region is the *Ovophis* species complex, which comprises at least five morphologically similar but distinct species, i.e., *Ovophis makazayazaya*, *Ovophis tonkinensis*, *Ovophis monticola*, *Ovophis zayuensis*, and *Ovophis convictus,* and, in addition, a long-recognized distinct islandic species, i.e., *Ovophis okinavensis* [11]. The former five species were considered representative of various geographical montane subspecies distributed across West Malaysia (Peninsular Malaya), Indochina (including Vietnam), the Indian subcontinent, southern China, and Taiwan Island, with some overlapping ranges. Locally, they are known by various names including the Indo-Malayan Mountain Pit Viper, Mountain Pit Viper, Blotched Pit Viper, Brown Pit Viper, and Mountain Iron-head Snake, all of which are distributed across Asia. The more easterly dispersed islandic population of *Ovophis okinavensis* (Hime Habu) is endemic to the Ryukyu Islands (including Okinawa) in the southern part of Japan [12]. Taking *O. convictus* from the Malayan Peninsula as an example, adult male snakes can grow up to 0.5 m while full-grown females may be over 1 m. The body is stout and the triangular head, which is blackish-brown in color, is relatively bigger than the neck. The small head and body scales are smooth, and the body is usually brown or greyish-brown with one or two dorsal series of large, squarish, dark-brown blotches (Figure 1). *Ovophis* pit vipers are ground-dwelling and terrestrial, and most of the montane species are found at elevations of over 700 m [12].

In the Malaysian experience, envenomation by *Ovophis* pit viper typically affects agricultural and rural communities in highland areas, in particular those in plantations and vegetable farms along the central mountain range of Peninsular Malaysia [13]. This phenomenon supports the notion that envenomation by these species is an occupational hazard to agricultural workers and their families residing near the habitat of the snake. Clinically, the envenomation caused by pit vipers can result in hemostatic derangement whereby victims develop hemorrhagic syndrome, characterized by prolonged bleeding time and coagulopathy [14,15]. Epidemiological data on envenomation caused by the *Ovophis* spp., however, is scarce in most parts of the world, presumably due to under-reporting. Bites were usually reported anecdotally or in isolated cases based on limited hospital records [14,16]. The venom of at least one of the species, *O. okinavensis*, demonstrated plasma procoagulant activity [17], while its lethal effect was approximately 3-fold weaker compared with the venom of *Protobothrops flavoviridis* (Okinawa Habu) [18]. Details of the venom properties, however, have not been investigated comprehensively and compared across different species. This hampers our understanding of the composition, antigenicity, and toxic activities of their venoms. Moreover, there is no specific antivenom available for this genus of medically important pit vipers, creating potentially life-threatening challenges for patients envenomed by these species, while the use of para-specific antivenom(s) as potential treatments has not yet been evaluated. Therefore, by applying a proteomic approach, this study aimed to unravel the venom composition of montane pit vipers from selected geographical origins. Hetero-specific antivenoms available in the region were also investigated for their immunological cross-reactivities toward the different *Ovophis* pit viper venoms. Given that coagulopathy is the predominant toxicity of *Ovophis* pit viper envenomation, the differential coagulotoxic activities of the venoms were studied, and the efficacy of the most immunoreactive hetero-specific antivenom in cross-neutralizing the toxicity was evaluated for insights into potential therapies.

## 2. Results and Discussion

### 2.1. Gel Electrophoretic Profiling of Ovophis Pit Viper Venoms

**Figure 2:**
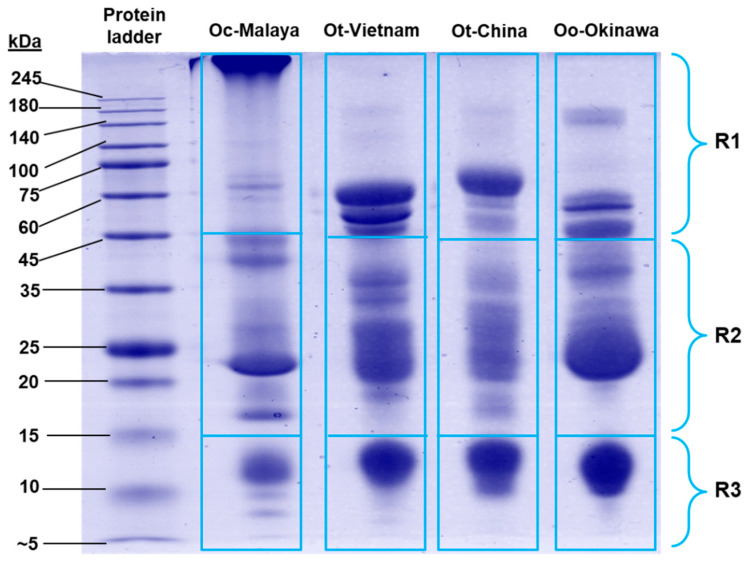
Sodium dodecyl sulfate-polyacrylamide gel electrophoresis of *Ovophis* pit viper venoms under reducing conditions. Proteins migrated on the gel were categorized into three molecular weight ranges, R1–R3 (<15 kDa, 15–45 kDa, and >45 kDa, respectively), and the gel band intensities were estimated with Pierce myImage Analysis Software. Abbreviations: Oc-Malaya, *Ovophis convictus* (West Malaysia); Ot-Vietnam, *Ovophis tonkinensis* (northern Vietnam); Ot-China, *Ovophis tonkinensis* (southern China); Oo-Okinawa, *Ovophis okinavensis* (Okinawa, Japan).

On SDS-PAGE, *Ovophis* venom proteins were separated according to molecular weights under reducing conditions (Figure 2). The protein composition of each venom was heterogenous, with multiple protein bands of varying intensity distributed across a wide range of molecular weights. The majority of proteins in the venoms were between 15 and 45 kDa, constituting 47.27–58.38% of total venom proteins (estimated by relative gel band intensity) (Supplementary Table S1). Along with the high molecular weight (>45 kDa) proteins, variable banding patterns were observed in SDS-PAGE of the different venoms. Meanwhile, the low molecular weight proteins (between 10 and 14 kDa) were present more uniformly across the samples.

### 2.2. Venom Proteomics of Ovophis Pit Viper Venoms

**Figure 3:**
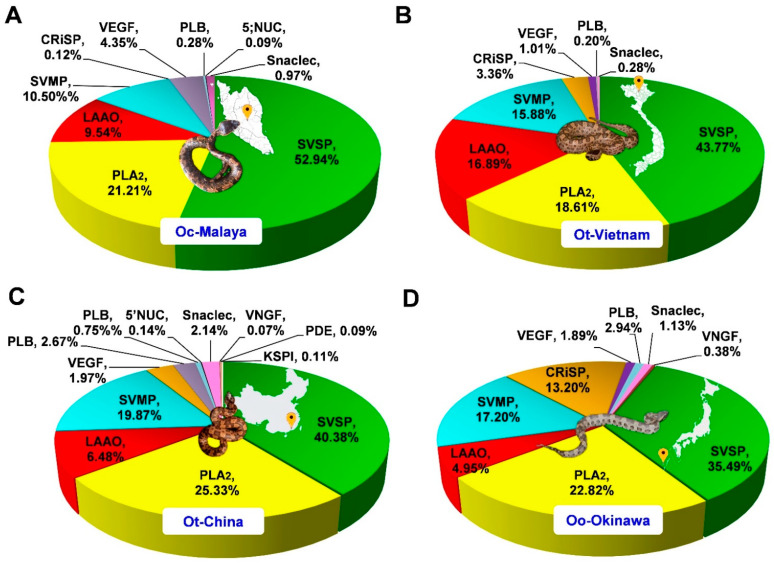
Venom proteomes of *Ovophis* pit viper venoms. (**A**) *Ovophis convictus* from West Malaysia (Oc-Malaya), (**B**) *Ovophis tonkinensis* from North Vietnam (Ot-Vietnam), (**C**) *Ovophis tonkinensis* from Southern China (Ot-China), (**D**) *Ovophis okinavensis* from Okinawa, Japan (Oo-Okinawa). Relative abundance is expressed as percentage of total venom proteins for individual protein families. Abbreviations: SVSP, snake venom serine proteases; PLA_2_, phospholipase A_2_; SVMP, snake venom metalloproteinase; LAAO, L-amino acid oxidase; Snaclec, snake venom C-type lectin; CRiSP, cysteine-rich secretory venom protein; VEGF, venom endothelial growth factor; PDE, phosphodiesterase; PLB, phospholipase B; 5′NUC, 5′ nucleotidase; VNGF, venom nerve growth factor; KSPI, Kunitz-type protease inhibitor. Photo credits: Choo Hock Tan (Ot-Malaya), Henry Lee (Ot-Vietnam), Abdel Bizid (Ot-China), Wikimedia (Oo-Okinawa).

The venom proteins were identified and analyzed by nano-LC-MS/MS, followed by data mining. The venom proteomes were assembled and sorted by the toxin families, as shown in Figure 3. Four major toxin protein families were identified in the *Ovophis* pit viper venom proteomes, i.e., snake venom serine protease (SVSP), phospholipase A_2_ (PLA_2_), snake venom metalloproteinase (SVMP), and L-amino acid oxidase (LAAO). SVSP were the most diversely and abundantly expressed proteins in all venoms, comprising multiple distinct forms and constituting approximately 35–53% of total venom proteins (Table 1). PLA_2_ were the second most abundant proteins in all venom proteomes, representing 21.21%, 18.61%, 25.33%, and 22.82% of total proteins in the venoms of Oc-Malaya, Ot-Vietnam, Ot-China, and Oo-Okinawa, respectively. This was followed by SVMP and LAAO, with protein abundances varying between 4.95% and 19.87% in the four snake venom proteomes. Other proteins detected were mostly of lower abundances, including families of cysteine-rich secretory proteins, venom endothelial growth factor, phospholipase B-like protein, 5′ nucleotidase, snaclecs, venom nerve growth factor, phosphodiesterase, and Kunitz-type serine protease inhibitor (Figure 3). Mass spectrometry results of the tryptic peptides for protein identification (including protein scores, mass-to-charge ratios, spectral counts and intensities, and sequences) are provided in Supplementary File S2.

In all four *Ovophis* pit viper venom proteomes, SVSP made up virtually half the bulk of proteins, indicating their crucial role in the biological activity of the venom. The domination of SVSP in these venoms was consistent with a previously reported venom-gland transcriptomic study of *O. okinavensis* which demonstrated that SVSP transcripts were highly expressed, accounting for as much as 93.11% of toxin transcription in the transcriptome [19]. SVSPs are classified within the clan PA as subclan S, family S1 (chymotrypsin), or subfamily A of the proteolytic enzymes (MEROPS classification, http://merops.sanger.ac.uk, accessed on 31 May 2021). Such proteases are characterized by a typical chymotrypsin fold and two six-stranded β-barrels, with active sites that lie in the cleft between the latter and include the canonical catalytic triad His-Asp-Ser [20]. SVSPs demonstrate substrate specificity and thus vary in their pharmacological activities, with virtually all affecting hemostasis. A number of SVSPs have only fibrinogenolytic activity and are called ‘thrombin-like’ enzymes for their ‘fibrinogen clotting’ activity [21,22]. Snake venom thrombin-like serine proteases preferentially release fibrinopeptide A and/or fibrinopeptide B from fibrinogen to produce friable fibrin clots composed of short polymers that are rapidly dispersed and no longer cross-linked by activated factor XIII [23]. The continuous event exhausts the normal fibrinogens in the plasma, resulting in consumptive coagulopathy and, consequently, systemic bleeding, hypovolemic shock, and death [15]. A few SVSPs, known as kallikrein-like proteases have the ability to release bradykinin from kininogen, and may cause a reduction in blood pressure as well as the inhibition of blood coagulation in victims [21]. In the *Ovophis* pit viper venoms, the diversity of SVSPs expressed implies possible deployment of different mechanisms in the pathogenesis of coagulopathy. Further studies should aim to delineate the substrate specificity and structure–activity relationship of the individual proteins. In this regard, *de novo* venom-gland transcriptomics and genomics of the different *Ovophis* pit vipers should be the subject of future research to unveil the authentic sequences of the diverse SVSPs belonging to each species. This will also provide crucial insights into the evolutionary significance of their venom phenotypes, in which SVSPs dominate the expression of toxins.

PLA_2_ present in the *Ovophis* pit viper venom proteomes were, in majority, matched by homology to mountain or highland pit vipers in Asia, e.g., *Ovophis* species (including *Ovophis makazayaya*), *Trimeresurus sabahi*, and *Trimeresurus cardamonensis*. PLA_2_ with sequences specific to *O. tonkinensis* in the database were identified in both Ot-Vietnam and Ot-China venoms, while PLA_2_ specific to *O. convictus* was matched to Oc-Malaya, besides other PLA_2_ proteoforms. Snake venom PLA_2_ are known to exhibit a wide array of pharmacological activities, but the major putative functions in the context of *Ovophis* pit viper envenomation are likely related to anticoagulant and proinflammatory effects [24,25]. All subtypes identified in the proteomes belonged to Group II secretory PLA_2_ typically present in viperid snake venoms. The PLA_2_ forms to which they were matched are of acidic type (theoretical pI 4–5) and contained aspartic acid as the 49th amino acid residue, which is critical for enzymatic activity (phospholipid hydrolysis) [26]. The catalytically active viperid D49 PLA_2_ are usually more prevailing in anticoagulant activity compared with the non-catalytic variants of myotoxic and/or neurotoxic K49 PLA_2_ [27].

LAAO is another protein family which may contribute to significant inflammatory and cytotoxic activities of the venom, with effects seemingly related, at least in part, to hydrogen peroxide, a secondary product formed during the chemical reaction catalyzed by LAAOs [28,29]. The number of LAAO forms detected varied from one to seven among the different venoms, implying potentially divergent LAAO functions in the different lineages. Snake venom LAAO proteins are known to be heavily glycosylated, resulting in glycoforms with varying physicochemical properties [30]. The proteomic approach per se, however, is limited in regard to profiling the glycosylation of LAAO. Glycomic and glycoproteomic studies would be needed in this context to shed light on the diversity of glycosylated LAAO.

While SVMPs are generally abundantly present in the venom proteomes of Asiatic viperids (for examples see [31,32,33]), the abundances of this enzymatic protein family in the *Ovophis* pit viper venoms studied were relatively low (<20% of total venom proteins). The majority of SVMP detected belong to Class P-III of SVMP, which are high molecular weight proteins containing disintegrin-like and cysteine-rich domains following the ADAM proteinase domain [34,35]. The structural complexity of P-III enzymes allows more diverse functions including hemorrhagic, proinflammatory, cytotoxic, procoagulant, and platelet aggregation-inhibiting activities, which are relevant in the context of *Ovophis* pit viper envenomation, although the relatively lower SVMP abundance likely indicates a lesser role in comparison with the dominant SVSP in the venoms. In the previously reported *O. okinavensis* venom-gland transcriptomics, SVMP transcripts likewise showed a low abundance of 4.2% toxin transcript abundance, with P-III transcripts being more abundantly expressed than P-II transcripts, while P-I was not detected [19]. This finding is largely in agreement with the present proteomic finding on *Ovophis* pit viper venoms.

Other venom proteins detected were variably low in abundances (<5% of total venom proteins), with the exception of CRiSP (13.2%) in *O. okinavensis* venom. Some of the CRiSPs derived from snake venoms are L-type Ca^2+^ or cyclic nucleotide-gated channel-blocking toxins, but their roles in snakebite envenomation remain largely unknown [36]. VEGFs may exhibit proinflammatory activity and cause vascular permeability increment [37], while snaclecs including C-type lectins can modulate and compromise platelet function [38], thus potentiating the hemotoxic effect of the envenomation. PLB-like proteins are probably the least investigated snake venom toxins; earlier studies suggested that this enzyme exhibited in vitro hemolytic activity [39,40]. Other proteins present in trace amounts were more variably detected in different venom samples. 5′NT (detected in Oc-Malaya and Ot-China), VNGF (detected in Ot-China and Oo-Okinawa), and PDE (detected in Oc-China) may induce local vasodilation and facilitate venom spread from the bite site [41,42]. KSPI was solely found in the venom of Ot-China with a negligible abundance, although this protein may induce *in vivo* anticoagulation [43]. These minor proteins, present in small amounts in the venoms, are usually not the lethal or principal venom components. Nevertheless, given their almost ubiquitous occurrence and evolutionary conservation in snake venoms, these proteins may play ancillary roles which are integral to the species’ survival and are thus worth investigating.

### 2.3. Immunoreactivity of Antivenoms toward Ovophis Pit Viper Venoms

**Figure 4:**
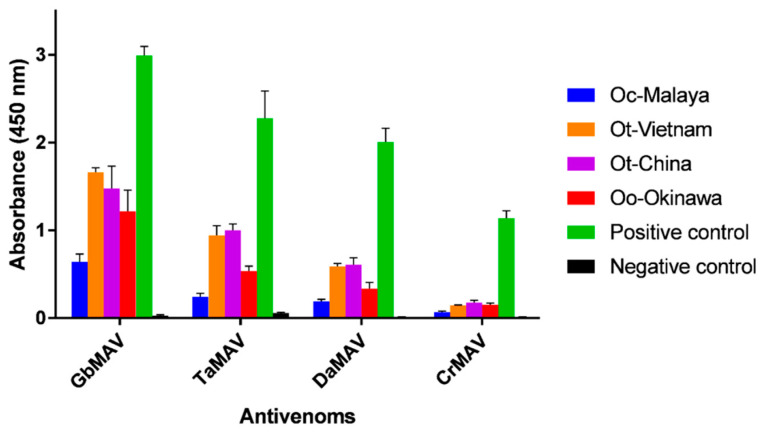
Immunological binding activity of four antivenoms toward *Ovophis* venoms. The antivenom dose used was 1:3000 dilution from a stock of 10 mg/mL. The absorbance values are means ± S.E.M. of triplicates. The positive and negative controls of the respective antivenoms used were as stated in Materials and Methods. Abbreviations: Oc-Malaya, *Ovophis convictus* from West Malaysia; Ot-Vietnam, *Ovophis tonkinensis* from northern Vietnam; Ot-China, *Ovophis tonkinensis* from southern China; Oo-Okinawa, *Ovophis okinavensis* from Okinawa, Japan.

Considering that there is neither species-specific nor genus-specific antivenom available against *Ovophis* pit vipers, four heterologous monovalent antivenom products (related to pit vipers) available in the regions of Southeast and East Asia were evaluated for their cross-reactivities. On the whole, the antivenoms showed limited immunoreactivity toward the *Ovophis* pit viper venoms compared with the respective homologous venoms (Figure 4). Among the different antivenoms, GbMAV and TaMAV were more immunoreactive than DaMAV and CrMAV by a approximately two to three-folds. This indicated that the venom antigenicity of *Ovophis* pit vipers was more similar to those of *G. brevicaudus* and *T. albolabris* than *D. acutus* and *C. rhodostoma*, which are phylogenetically less related basal crotalids [44].

**Figure 5:**
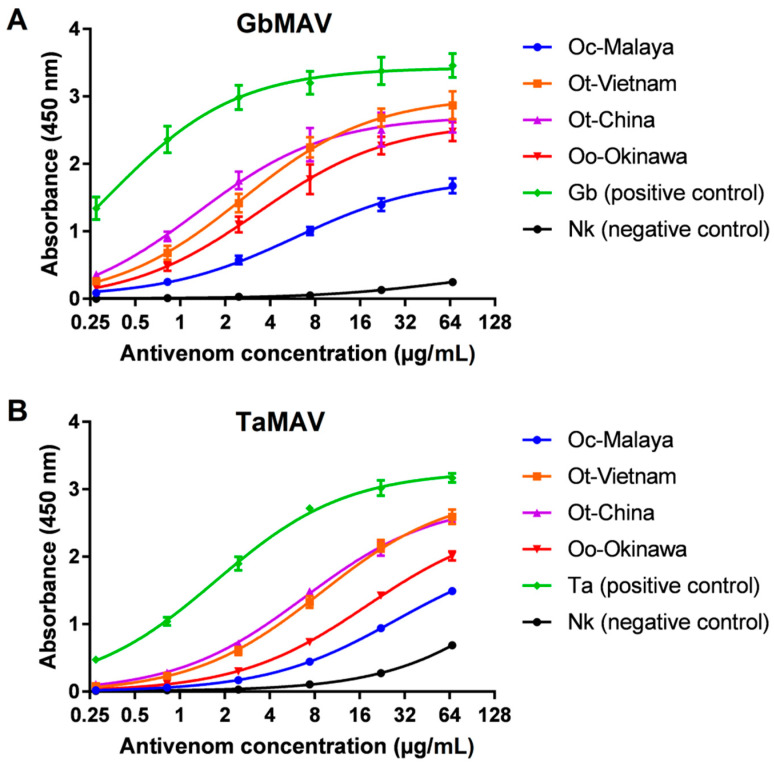
Concentration-dependent immunoreactivity of antivenoms toward *Ovophis* pit viper venoms. (**A**) Chinese *Gloydius brevicaudus* monovalent antivenom (GbMAV), (**B**) Thai *Trimeresurus albolabris* monovalent antivenom (TaMAV). Absorbance values are means ± S.E.M. of triplicates. Abbreviations: Oc-Malaya, *Ovophis convictus* from West Malaysia; Ot-Vietnam, *Ovophis tonkinensis* from northern Vietnam; Ot-China, *Ovophis tonkinensis* from southern China; Oo-Okinawa, *Ovophis okinavensis* from Okinawa, Japan; Ta, *Trimeresurus albolabris*; Nk, *Naja kaouthia*.

The immunological binding activities of GbMAV and TaMAV were then examined at serial concentrations. The two antivenoms showed increasing immunoreactivity in a dose-dependent manner toward all *Ovophis* pit viper venoms tested, with GbMAV appearing to be more efficacious than TaMAV in binding (Figure 5). The serial immunoreactivities of GbMAV toward Ot-Vietnam, Ot-China, and Oo-Okinawa were comparable, while its immunoreactivity was significantly lower toward Oc-Malaya, with a higher half-maximal concentration (EC_50_) (Table 2). The immunoreactivities of TaMAV toward Ot-Vietnam and Ot-China were comparable, low toward Oo-Okinawa, and much lower toward Oc-Malaya. The finding suggests that Ot-Vietnam and Ot-China venoms shared rather conserved protein epitopes despite their separated geographical origins, and this is, in a way, reflective of the single species status for the two according to the revised taxonomy proposed by Malhotra et al. [11]. Of note, the southern and southwestern Chinese species was previously known as *Ovophis orientalis*, which was regarded to be distinct from *O. tonkinensis* in northern Vietnam. Comparing Ot-Vietnam, Ot-China, and Oo-Okinawa, all three venoms were antigenically similar to the *Gloydius* species, while Oo-Okinawa was less antigenically related than the former two to *Trimeresurus* species. Phylogenetically, *O. okinavensis* sits basal to *Gloydius* rather than being closely related to other *Ovophis* species and *Trimeresurus* pit vipers [44]. On the other hand, both antivenoms showed markedly lower immunoreactivities toward Oc-Malaya venom, suggesting that the venom of the *O. convictus* from West Malaysia is antigenically varied from Ot-Vietnam, Ot-China, and Oo-Okinawa from the northern subtropics closer to the Tropic of Cancer. The interspecies discrepancy suggests a possible venom phenotypic variation associated with the geographical distribution of Oc-Malaya, being further to the south near the Equator where a tropical climate predominates.

On the other hand, all *Ovophis* pit viper venoms showed weak-to-moderate binding with the antivenoms tested, in comparison with the homologous venoms used in the production of the respective products (Figure 5). The immunological cross-reactivity of antivenom toward venoms of different genera is anticipated to be low, since venom compositions are likely to vary significantly across different genera of snakes. Apparently, while *Ovophis* pit viper venoms were characterized by highly abundant SVSP and relatively less SVMP, the converse was observed in the venom proteomes of *G. brevicaudus*, *T. albolabris*, *D. acutus*, and *C. rhodostoma*, in which a higher proportion of SVMP instead of SVSP was reported [31,45,46,47]. The remarkable differences in venom proteomes are likely accompanied by variations in the overall antigenicity of the venom proteins. Moreover, SVSP (the predominant toxins in *Ovophis* venoms) may have glycan chains that can alter the surface epitope and thus compromise the immunoreactivity of the antivenoms [48,49].

### 2.4. Procoagulant Effect of Ovophis Venoms and Its Neutralization by Antivenoms

Existing immunoassays including ELISA, immunoblotting, and affinity chromatography-based antivenomics elucidate the immunological binding activity of antivenom to venom proteins, but these assays do not determine if the antivenom is functionally effective in neutralizing the pathological activities of the venom. Hence, the neutralizing efficacy of GbMAV and TaMAV were further evaluated against the procoagulant activity of *Ovophis* pit viper venoms in human plasma. The procoagulant activity is putatively induced by the principal toxin SVSP, which dominated the venom proteomes (Figure 3) and is responsible for venom-induced consumptive coagulopathy in most envenomation caused by pit vipers, including clinically observed envenomation by *Ovophis* spp. [14,15]. Tested in human plasma, the venoms showed moderate-to-strong procoagulant activity (minimum coagulant dose, MCD = 4–10 µg/mL), with differential potency in the decreasing order of Ot-Vietnam > Oo-Okinawa > Oc-Malaya (Table 3). The venom of Ot-China, on the other hand, demonstrated a remarkably higher procoagulant activity in which its MCD was estimated to be lower than 1 µg/mL. In the *Ovophis* pit viper venoms, the plasma-clotting effect is consistent with the dominance of SVSP in their proteomes, as also observed in several Asiatic pit vipers which have convergently evolved the coagulopathic mechanism, causing similar hemotoxic manifestations in envenomation [31,32,50]. Different toxin components in the respective venoms, however, may have varying potency and synergistic interaction. Further works with purification and characterization of the individual procoagulant toxins will be needed to elucidate the differential potency of venom procoagulant activity.

In the neutralization assay, GbMAV and TaMAV were able to mitigate the procoagulant effects of the venoms to varying degrees. The cross-neutralization effects of GbMAV and TaMAV were generally comparable, with both being most efficacious against the Ot-Vietnam venom (effective dose, ED, of approximately 3 µL), while efficacy against Oo-Okinawa and Oc-Malaya were moderate (ED of approximately 5–6 µL) (Table 3). The cross-neutralization finding is corroboratively supported by the immunological cross-reactivity, which indicated that GbMAV and TaMAV bound to most of the procoagulant toxins in the venoms, thus inhibiting the coagulotoxic effect. The efficacy of the cross-neutralization, however, might not be congruent with the degree of immunoreactivity, as the immunoreactivity was a reflection of antivenom binding to all antigenic proteins including nontoxic components in the venom. The functional neutralization is therefore a more robust and valid assessment for antivenom efficacy and is especially essential when exploring the therapeutic potential of hetero-specific antivenoms. The current finding suggests that both GbMAV which is available in China, and TaMAV in Southeast Asia, are potentially alternative choices of antivenom that may be repurposed to treat the coagulopathic envenomation caused by *Ovophis* pit vipers, pending dosage optimization and clinical trials.

On a side note, the neutralization of venom-induced lethality is normally a standard test of antivenom efficacy [51,52]; however, the *Ovophis* pit viper venoms were not found to be lethal to mice at a high dose of 4 µg/g in the current work. The lack of venom lethality is clinically possible, as fatality due to envenomation by these species has not been reported, although patients can develop hemotoxic complication with consumptive coagulopathy which is potentially life-threatening without proper treatment.

## 3. Conclusions

The present study characterized the venom proteomes of three *Ovophis* pit viper species from four geographical locales, demonstrating protein compositions that were dominated by SVSP (40–60% of total venom proteins) with putative procoagulant activity. The venoms induced moderate-to-strong clotting effect in citrated human plasma, indicating the role of consumptive coagulopathy in the pathophysiology of envenomation. The hetero-specific antivenoms GbMAV and TaMAV were both immunoreactive toward the venoms, and were variably effective in cross-neutralizing their procoagulant activities. Taken together, the study suggests the potential para-specific utility of the antivenoms in the treatment of coagulotoxic envenomation caused by *Ovophis* pit vipers in their respective regions.

## 4. Methods and Materials

### 4.1. Venoms and Antivenoms

Venom samples of *Ovophis* species complex were sourced from specimens originated from West Malaysia (*O. convictus*), northern Vietnam, the southern Chinese mainland (*O. tonkinensis*), and Okinawa Island of Japan (*Ovophis* *okinavensis*). For simplicity, the venom samples were labeled with abbreviations in this report as follows: Oc-Malaya, Ot-Vietnam, Ot-China, Oo-Okinawa, respectively, as per above. Venoms were stored in lyophilized form at −20 °C until use. Other samples used as controls in the immunoreactivity study were venoms of *Naja kaouthia*, *Trimeresurus albolabris*, and *Calloselasma rhodostoma*, supplied by the Queen Saovabha Memorial Institute (QSMI, Bangkok, Thailand), and *Deinagkistrodon acutus, Gloydius brevicaudus*, and *Protobothrops mucrosquamatus*, obtained from Latoxan (Valence, France).

The hetero-specific antivenoms used in the present study were: (1) *Gloydius brevicaudus* monovalent antivenom (GbMAV, liquid antivenom, raised against *G. brevicaudus* venom of Chinese origin, batch no. 20141001); (2) *Deinagkistrodon acutus* monovalent antivenom (DaMAV, liquid antivenom, raised against *D. acutus* venom of Chinese origin, batch no. 20140501); (3) green pit viper (*Trimeresurus albolabris*) monovalent antivenom (TaMAV, lyophilized antivenom, raised against Thai *T. albolabris* venom, batch no. TA00116); (4) *Calloselasma rhodostoma* monovalent antivenom (CrMAV, lyophilized antivenom, raised against Thai *C. rhodostoma* venom, batch no. TA00116). Lyophilized antivenoms were reconstituted in 10 mL of ultrapure water prior to use as per the manufacturer’s instructions. Protein concentration of all antivenoms was determined using Pierce Bicinchoninic Acid Protein Assay Kit (ThermoFisher Scientific, Waltham, MA, USA).

### 4.2. Chemicals and Materials

All chemicals and reagents used were of analytical grade. Ammonium bicarbonate, dithiothreitol (DTT), and iodoacetamide were purchased from Sigma-Aldrich (St. Louis, MO, USA). MS-grade trypsin protease was purchased from ThermoFisher Scientific (Waltham, MA, USA). Millipore ZipTip C_18_ pipette tips were purchased from Merck (Kenilworth, NJ, USA). PM2700 ExcelBand 3-color Broad Range Protein Marker (~5–245 kDa) was obtained from SMOBIO (Hsinchu, Taiwan).

### 4.3. Sodium Dodecyl Sulfate-Polyacrylamide Gel Electrophoresis (SDS-PAGE)

Forty micrograms of the respective *Ovophis* venoms were electrophoresed with 15% SDS-PAGE under reducing conditions in accordance with the Laemmli protocol [53], calibrated with the prestained protein ladder (5–245 kDa). The electrophoretic separation was conducted at 90 V for 120 min. Proteins were stained with Coomassie Brilliant Blue R-250 for visualization. The gel was then scanned using Image Scanner III Labscan 6.0 (GE Healthcare, Chicago, IL, USA). The relative intensity of gel regions (I, II, and III) were determined using myImage analysis software (Thermo Scientific, Waltham, MA, USA).

### 4.4. In-Solution Tryptic Digestion and Tandem Mass Spectrometry (Nano-ESI-LCMS/MS)

Ten micrograms of each venom sample of *Ovophis* species were subjected to reduction with DTT and alkylation with iodoacetamide, and were digested in-solution with mass spectrometry-grade trypsin proteases as described previously [54]. The digested peptides were desalted and extracted with Millipore ZipTip C_18_ pipette tips in preparation for mass-spectrometry analysis.

For protein identification, the extracted peptides were reconstituted in 7 μL of 0.1% formic acid in water. Peptides were subjected to nano-electrospray ionization-liquid chromatography-tandem mass spectrometry (nano-ESI-LCMS/MS) with an Agilent 1200 HPLC-Chip/MS Interface coupled with the Agilent 6520 Accurate-Mass Q-TOF LC/MS system. The peptides were loaded (1 μL) in a 300 Å, C_18_ enrichment column, followed by a 75 μm × 150 mm analytical column (Agilent No.: G4240-62010). Solvent A (0.1% formic acid in water) and solution B (90% acetonitrile in water with 0.1% formic acid) were used for elution of the peptides at the stepwise linear gradients: 3–50% solution B for 30 min, 50–95% solution B for 2 min, and 95% solution B for 5 min. The ion polarity was set to positive ionization polarity mode. Positive ionization mode was selected for ion polarity. Flow rate and temperature of drying gas were set to 11 L/min and 290 °C, respectively. Fragmentor voltage was configured to 175 V and the capillary voltage was set to 1800 V. Spectra were obtained in MS/MS mode, with an MS scan range of 200–3000 *m/z* and MS/MS scan range of 50–3200 *m/z*. Precursor charge selection was set as doubly charged state and above, with the exclusion of precursors 1221.9906 *m/z* (z = 1) and 299.2944 (z = 1) set as reference ions. Data were generated with MH (protonated peptide ion) mass span between 50 and 3200 Da and sorted with Agilent Spectrum Mill MS Proteomics Workbench software packages version B.06.00.201. Carbamidomethylation of cysteine residues was set as a fixed modification and oxidation of methionine residues as a variable modification. The spectra were searched against a nonredundant protein sequence database from the National Center for Biotechnology Information (NCBI) (taxonomy: Serpentes, taxid: 8570). The identified proteins or peptides were validated with the following filters: protein score >10, peptide score >10, and scored peak intensity (SPI) >70%. The relative abundance of each protein was estimated based on the ratio of mean spectral intensity (MSI) of the protein relative to the total MSI of all proteins identified within a molecular weight range indicated by SDS-PAGE. The MSI ratio of the protein was then multiplied by the gel band intensity accordingly, with the calculation formula as shown below:

$$\begin{array}{c} \begin{array}{c} \;\mathrm{Relative}\;\mathrm{abundance}\;\mathrm{of}\;\mathrm{Protein}\;A\;\left(\%\right)\\ \;=\;\frac{\mathrm{Mean}\;\mathrm{spectral}\;\mathrm{intensity}\;(\mathrm{MSI})\mathrm{of}\;\mathrm{Protein}\;A\;\mathrm{in}\;\mathrm{SDS}\;\mathrm{PAGE}\;\mathrm{region}\;I}{\mathrm{Total}\;\mathrm{MSI}\;\mathrm{of}\;\mathrm{proteins}\;\mathrm{in}\;\mathrm{SDS}\;\mathrm{PAGE}\;\mathrm{region}\;I}\\ \;\times \;\mathrm{Gel}\;\mathrm{band}\;\mathrm{intensity}\;\mathrm{of}\;\mathrm{SDS}\;\mathrm{PAGE}\;\mathrm{region}\;I\;(\%) \end{array} \end{array}$$

### 4.5. Data Availability

The mass spectrometry data have been deposited to the ProteomeXchange Consortium (http://proteomecentral.proteomexchange.org, dataset uploaded on 1 June 2021) via the iProX partner repository [55] with the dataset identifier PXD: PXD026416.

### 4.6. Enzyme-Linked Immunosorbent Assay (ELISA)

The immunoreactivity of the antivenoms toward the four *Ovophis* venoms was examined through an indirect enzyme-linked immunosorbent assay (ELISA), optimized in the laboratory [56]. *N. kaouthia* venom was used as a negative control for all assays. Positive controls were the venoms of *G. brevicaudus* (for GbMAV), *D. acutus* (for DaMAV), *T. albolabris* (for TaMAV), and *C. rhodostoma* (for CrMAV), respectively. Briefly, 96-well immunoplates were coated overnight with 10 ng of *Ovophis* venom in 100 µL coating buffer at 4 °C. The plates were washed four times with phosphate-buffered saline (Oxiod, Hampshire, UK) containing 0.5% Tween 20 (Sigma-Aldrich, St. Louis, MO, USA) (PBST). Then, 100 µL of each antivenom dilution (1:3000) from a stock of 10 mg/mL were added to each well and incubated for one hour at room temperature. The wash step was repeated and 100 µL of 1:10,000 diluted horseradish peroxidase-conjugated anti-horse IgG (Jackson ImmunoResearch Inc., West Grove, PA, USA) in PBST was added to each well and incubated for 1 h at room temperature. After another cycle of washing, 50 µL of TMB substrate (3,3′,5,5′-Tetramethylbenzidine) solution was added to each well and incubated in the dark for 25 min at room temperature. Finally, 50 µL of 12.5% sulfuric acid was added to terminate the reaction before the absorbance reading at 450 nm. The experiments were performed in triplicate and absorbance values were expressed as means ± S.E.M.

Following this, the two monovalent antivenoms (GbMAV and TaMAV) which showed relatively high immunoreactivity toward *Ovophis* venoms were further examined in the concentration-dependent study. Serially diluted antivenoms (1:150, 1:450, 1:1350, 1:4050, 1:12,150, and 1:36,450), prepared in 100 µL from a stock of 10 mg/mL, were used. All procedures of indirect ELISA proceeded as described above. The half-maximal effective concentration (EC_50_, in µg/mL), defined as the antivenom dose at which half-maximal binding occurred, was determined using GraphPad Prism (version 7.04).

### 4.7. Procoagulant Activity of Venom and Neutralization by Antivenom

The procoagulant activity of *Ovophis* venoms was assessed with a modified turbidimetric method using citrated human plasma as previously described [57]. In brief, 100 μL of citrated human plasma, containing 0.4 M CaCl_2,_ was added to 100 μL of venom of various concentrations in a 96-well microplate at 37 °C. The procoagulant activity was assessed by monitoring absorbance at 405 nm for 15 min, with readings taken in 15 s intervals, using the Infinite M1000 Pro Multimode plate reader (Männedorf, Switzerland). An increase in the absorbance by 0.02 units from the mean of the two preceding absorbance measurements indicates plasma clotting. The minimum clotting dose (MCD) was defined as the minimal venom dose (µg) required to induce clotting of plasma in 5 min.

The neutralization of procoagulant activity was conducted by incubating a venom dose of 2MCD of respective venoms with different doses of antivenom (GbMAV and TaMAV), to make up a total volume of 100 µL, at 37 °C for 30 min. Following that, the mixture was added to 100 μL of citrated human plasma, containing 0.4 M CaCl_2_. The absorbance was monitored and the clotting time was determined as described above. The effective dose (ED) of antivenom was defined as the dose of antivenom (µL) required to prolong the clotting time of the plasma by three times that of the control (2MCD with no antivenom).

## Figures and Tables

**Table 1:** Snake venom protein families and subtypes in *Ovophis* pit viper venoms.

Protein Family and Subtype	Accession No.	Relative Protein Abundance in Percentage (% of Total Venom Proteins). Bracket Indicates the Number of Homologous Subtypes Identified.			
		Oc-Malaya (40)	Ot-Vietnam (49)	Ot-China (62)	Oo-Okinawa (39)
**Snake Venom Serine Protease**		**52.94 (18)**	**43.77 (22)**	**40.38 (22)**	**35.49 (19)**
Serine endopeptidase	A0A2I7YS44	1.65	1.90	-	-
Serine endopeptidase	A0A2I7YS46	-	-	1.38	1.36
Serine endopeptidase	A0A2I7YS67	-	-	-	1.40
Serine endopeptidase	A0A2I7YS70	-	0.59	3.74	-
Serine protease	A0A1Y0DIB4	2.20	-	-	-
Serine protease	U3TBG1	2.03	-	-	-
Serine protease VLSP-3	E0Y420	3.02	-	1.43	2.44
Serine proteinase 1	A0A194AM91	1.99	2.10	-	-
Serine proteinase 10	A0A194ARW1	-	0.63	-	-
Serine proteinase 10b	A0A194ARP9	2.75	-	-	-
Serine proteinase 11a	A0A194ATG3	-	1.97	-	-
Serine proteinase 12a	A0A1W7RJV2	6.83	3.41	0.34	
Serine proteinase 14a	A0A194APL6	-	-	-	1.49
Serine proteinase 15a	A0A1W7RJS4	-	-	2.41	-
Serine proteinase 16	A0A194APB8	-	2.75	-	-
Serine proteinase 19b	A0A194APB3	2.89	4.70	-	-
Serine proteinase 1a	A0A0K8RS97	-	-	1.70	-
Serine proteinase 1b	T1D6M9	-	2.29	-	-
Serine proteinase 1b	T1E3X7	-	-	-	0.67
Serine proteinase 2	A0A194ARH4	-	1.61	1.66	-
Serine proteinase 9	A0A1W7RJU0	1.87	-	0.31	-
Serine proteinase 9b	A0A194ASS1	5.90	1.66	1.48	-
Serine proteinase 9c	A0A194AS00	-	-	-	0.90
Snake venom serine protease	A0A194ALW4	-	-	-	1.04
Snake venom serine protease	A0A194AM96	-	-	-	1.85
Snake venom serine protease	A0A194APB3	-	-	1.88	1.62
Snake venom serine protease	A0A194APJ3	-	2.90	-	2.76
Snake venom serine protease 1	O13059	-	1.03	-	-
Snake venom serine protease 2C	O13062	-	-	1.50	-
Snake venom serine protease homolog KN4	Q71QJ4	-	-	3.86	-
Snake venom serine protease HS112	Q5W960	-	-	1.76	-
Snake venom serine protease pallabin	Q9YGJ2	-	-	-	1.17
Snake venom serine protease pallase	O93421	-	2.64	-	-
Thrombin-like enzyme AhV_TL-I	I4CHP3	4.00	-	-	1.43
Thrombin-like enzyme asperase	Q072L6	2.97	-	-	-
Thrombin-like enzyme bilineobin	Q9PSN3	2.23	2.56	-	2.96
Thrombin-like enzyme collinein-4	C0HK18	-	-	0.83	-
Thrombin-like enzyme contortrixobin	P82981	4.72	3.23	1.67	4.50
Thrombin-like enzyme elegaxobin-1	P84788	1.67	1.21	1.55	-
Thrombin-like enzyme halystase	P81176	-	-	1.50	-
Thrombin-like enzyme kangshuanmei	P85109	2.55	-	2.55	-
Thrombin-like enzyme kangshuanmei	P85109	-	-	-	-
Thrombin-like enzyme stejnobin	Q8AY81	1.46	0.06	-	-
Alpha-fibrinogenase shedaoenase	Q6T5L0	-	-	-	1.48
Bradykinin-releasing enzyme KR-E-1	Q7SZE2	-	-	-	3.11
Venom plasminogen activator 1	A0A286S0D8	-	-	3.30	-
Venom plasminogen activator 2	A0A286S0E6	-	2.38		-
Venom plasminogen activator LV-PA	Q27J47	-	-	1.96	-
Venom plasminogen activator TSV-PA	Q91516	-	-	1.64	1.99
Venom thrombin-like enzyme	A1E2S1	-	-	1.92	1.52
BATXSVSP11	A0A1L8D610	-	2.28	-	1.82
BATXSVSP14	A0A1L8D5V3	-	0.14	-	-
Cadam10_SVSP-12	A0A1W7RB66	2.21	1.73	-	-
**Phospholipase A_2_**		**21.21(3)**	**18.61(2)**	**25.33(7)**	**22.82 (3)**
Phospholipase A_2_	A0A0H3U1W3	-	-	4.87	-
Phospholipase A_2_	A0A0H3U1Y1	-	-	-	2.75
Phospholipase A_2_	A0A0H3U1Y3	-	15.80	5.28	-
Phospholipase A_2_	A0A0H3U1Z8	-	2.81	-	-
Phospholipase A_2_	A0A0H3U208	10.46	-	-	-
Phospholipase A_2_	A0A0H3U209	-	-	6.41	8.08
Phospholipase A_2_	A0A0H3U248	-	-	1.34	-
Phospholipase A_2_	U5HS18	7.64	-	-	-
Acidic phospholipase A_2_ 4	P81479	3.11	-	7.15	11.99
Acidic phospholipase A_2_ Drk-a1	A8CG86	-	-	0.18	-
Basic phospholipase A_2_ Drk-b1	A8CG89	-	-	0.11	-
**L-Amino-Acid-Oxidase**		**9.54(5)**	**16.89(7)**	**6.48(5)**	**4.95(1)**
Amine oxidase	A0A068EPZ2	1.40	4.31	1.21	-
Amine oxidase	T2HQ57	1.45	-	-	4.95
Amine oxidase	T2HRS5	-	2.63	1.04	-
L-amino acid oxidase	X2JCV5	4.37	-	2.02	-
L-amino-acid oxidase	A0A0K8RYS7	-	3.85	-	-
L-amino-acid oxidase	A6MFL0	-	0.38	-	-
L-amino-acid oxidase	O93364	1.07	1.27	-	-
L-amino-acid oxidase	P0DI84	1.26	-	-	-
L-amino-acid oxidase	P81382	-	0.19	-	-
L-amino-acid oxidase	Q4JHE3	-	4.27	0.99	-
L-amino acid oxidase bordonein-L	C0HJE7	-	-	1.23	-
**Snake Venom Metalloproteinase**		**10.50(5)**	**15.88(11)**	**19.87 (14)**	**17.20(5)**
**PII**					
Metalloprotease P-II 3	A0A077L6V8	-	-	0.13	-
p-ii_metalloprotease	U3TDH2	-	-	-	3.25
Zinc metalloproteinase/disintegrin	Q6T271	-	1.20	-	-
**PIII**					
Metalloprotease	A0A0C4ZNF1	0.87	-	-	-
Metalloprotease PIIa	V5IWE4	-	-	-	0.76
Metalloprotease PIII	V5IWF4	-	-	0.77	-
Metalloproteinase (Type III) 2b	A0A0K8RZ04	-	1.92	0.87	-
Metalloproteinase (Type III) 2b	J3RY86	-	-	1.80	-
Metalloproteinase (Type III) 5a	A0A0B8RV98	0.72	-	-	-
Metalloproteinase (Type III) 6a	A0A1W7RJU5	-	0.27	-	-
Metalloproteinase type III 10b	A0A194APP8	-	0.49	-	-
Metalloproteinase type III 12b	A0A194APP0	-	-	1.83	-
Metalloproteinase type III 13	A0A194ARL7	-	-	1.20	-
Metalloproteinase type III 2a	A0A194AMD0	-	0.33	0.40	-
p-iii_metalloprotease	U3TBS9	-	0.60	1.98	10.93
Snake venom metalloproteinase (Type III) 5	J3S831	-	-	1.57	-
Zinc metalloproteinase/disintegrin	P0C6E4	-	1.22	-	-
Zinc metalloproteinase-disintegrin-like daborhagin-K	B8K1W0	-	-	0.14	-
Zinc metalloproteinase-disintegrin stejnitin	P0DM87	0.13	-	2.95	1.85
Zinc metalloproteinase/disintegrin-like HR1a	Q8JIR2	8.13	5.40	4.56	-
Zinc metalloproteinase-disintegrin-like HR1b	P20164	-	1.35	0.39	-
Zinc metalloproteinase-disintegrin-like halysase	Q8AWI5	0.64	-	1.27	-
BATXSVMPII6	A0A1L8D5Z0	-	-	-	0.41
BATXSVMPIII8	A0A1L8D5Y0	-	0.76	-	-
Cadam10_SVMPIII-6	A0A1W7RB97	-	2.35	-	-
**Cysteine-Rich Venom Protein**		**0.12(1)**	**3.36(3)**	**1.97(2)**	**13.20(4)**
Cysteine-rich secretory protein 1c	A0A194APW7	-	-	-	3.14
Cysteine-rich secretory protein 1b	A0A194AQ87	-	0.13	-	-
Cysteine-rich secretory protein 1b	A0A194AS36	-	-	0.90	-
Cysteine-rich Venom Protein Moojin	A0A2H4N3D5	-	-	-	3.78
Cysteine-rich seceretory protein Bs-CRP	F2Q6E4	0.12	2.52	1.07	3.26
Cysteine-rich seceretory protein Ch-CRPKa	F2Q6E5	-	0.70	-	-
Cysteine rich secretory protein	T2HPR8	-	-	-	3.01
**Venom Endothelial Growth Factor**		**4.35 (2)**	**1.01(1)**	**2.67(1)**	**1.89(2)**
Snake venom vascular endothelial growth factor toxin	P67862	4.28	1.01	2.67	-
Vascular endothelial growth factor	A0A077L6N5	-	-	-	0.19
Vascular endothelial growth factor-like protein	T2HQ62	0.07	-	-	1.70
**Phospholipase-B-Like**		**0.28(2)**	**0.20(1)**	**0.75(2)**	**2.94(2)**
Phospholipase B-like	A0A077L7E7	-	0.20	-	-
Phospholipase B-like	A0A1W7RB94	0.16	-	0.40	-
Phospholipase B-like	T2HQ75	-	-	-	1.57
Phospholipase B-like	V8ND68	0.12	-	0.35	1.37
**5′ Nucleotidase**		**0.09(1)**	**-**	**0.14(1)**	**-**
Snake venom 5′-nucleotidase	B6EWW8	0.09		0.14	-
**Snaclec**		**0.97(3)**	**0.28(2)**	**2.14(5)**	**1.13(2)**
C-type lectin 2	A0A0K8RZ50	0.08	-	0.12	-
C-type lectin alpha subunit	T2HQM1	-	-	-	0.52
C-type lectin B subunit	A0A077L6M9	0.50	-	0.54	-
C-type lectin BPL	P0DL30	0.39	0.07	-	-
C-type lectin J	A0A0A1WDW9	-	-	0.13	-
Galactose binding lectin	T2HS62	-	-	-	0.61
Snaclec jerdonibitin subunit beta	D1MGU1	-	0.22	1.26	-
Snaclec rhodocetin subunit delta	D2YW40	-	-	0.09	-
**Venom Nerve Growth Factor**		**-**	**-**	**0.07(1)**	**0.38(1)**
Nerve growth factor	A0A077L854	-	-	0.07	-
Nerve growth factor	T2HPR2	-	-	-	0.38
**Phosphodiesterase**		**-**	**-**	**0.09 (1)**	**-**
Phosphodiesterase	A0A194AS02	-	-	0.09	-
**Kunitz-Type Serine Protease Inhibitor**		**-**	**-**	**0.11(1)**	**-**
Kunitz-type serine protease inhibitor 2	P00990	-	-	0.11	-

Abbreviations: Oc-Malaya, *Ovophis convictus* from West Malaysia; Ot-Vietnam, *Ovophis tonkinensis* from northern Vietnam; Ot-China, *Ovophis tonkinensis* from southern China; Oo-Okinawa, *Ovophis okinavensis* from Okinawa, Japan. ‘-’ indicates “Not detected”.

**Table 2:** Half-maximal concentrations (EC_50_) of *Gloydius brevicaudus* monovalent antivenom (GbMAV) and Thai *Trimeresurus albolabris* monovalent antivenom (TaMAV) toward *Ovophis* venoms.

Venom	GbMAV	TaMAV
	EC_50_ (µg/mL)	EC_50_ (µg/mL)
*Ovophis convictus* (West Malaysia)	5.90 ± 0.56	27.78 ± 0.20
*Ovophis convictus* (Vietnam)	2.60 ± 0.14	8.87 ± 0.62
*Ovophis convictus* (China)	1.31 ± 0.12	6.89 ± 0.38
*Ovophis okinavensis* (Japan)	3.34 ± 0.10	17.98 ± 0.480

Values are means ± S.E.M. of triplicates.

**Table 3:** Procoagulation and neutralization of *Ovophis* venoms by *Gloydius brevicaudus* monovalent antivenom (GbMAV) and *Trimeresurus albolabris* monovalent antivenom (TaMAV).

Venom	Procoagulant Activity		Antivenom Neutralization		
		GbMAV		TaMAV	
	MCD ^a^ (µg/mL)	ED ^b^		ED ^b^	
		µL	mg/mL	µL	mg/mL
Oc-Malaya	9.55 ± 0.75	4.81 ± 0.32	0.80	6.11 ± 0.76	0.63
Ot-Vietnam	4.25 ± 0.15	2.72 ± 0.42	0.63	3.06 ± 0.26	0.56
Oo-Okinawa	7.05 ± 0.05	6.13 ± 1.03	0.46	6.03 ± 0.44	0.47

GbMAV, *Gloydius brevicaudus* monovalent antivenom; TaMAV, *Trimeresurus albolabris* monovalent antivenom; MCD, minimum clotting dose; ED, effective dose. ^a^ MCD is the dose of venom (μg/mL) required to induce clotting of plasma in 5 min. ^b^ ED was defined as the dose of antivenom capable of prolonging the clotting time of the challenge dose (2 MCD) to 3 times that of the control, expressed in units of antivenom volume (μL) and venom amount per unit volume of antivenom (mg/mL). Values are indicated as means ± standard error of means (SEM) from three independent experiments. Protein concentrations: GbMAV (10.67 ± 0.50 mg/mL) and TaMAV (13.13 ± 0.98 mg/mL).

## Data Availability

The data presented in this study are openly available in ProteomeXchange Consortium (http://proteomecentral.proteomexchange.org, dataset uploaded on 1 June 2021) via the iProX partner repository with the dataset identifier PXD: PXD026416.

## Supplementary Materials

The following are available online at https://www.mdpi.com/article/10.3390/toxins13080514/s1, Table S1: Relative gel band intensity of *Ovophis* pit viper venoms, File S2: Mass spectrometry data of *Ovophis* pit viper venom proteomics.
