# Supplementary material for: Snake Venom Proteomics, Immunoreactivity and Toxicity Neutralization Studies for the Asiatic Mountain Pit Vipers, Ovophis convictus, Ovophis tonkinensis, and Hime Habu, Ovophis okinavensis
Source: Toxins (Basel). 2021 Jul 23;13(8):514. doi: 10.3390/toxins13080514 (PMC8402492; doi:10.3390/toxins13080514)
Supplement: Supplementary file 1 [file toxins-13-00514-s001.zip › Supplementary Table S1.pdf]

Snake Venom Proteomics, Immunoreactivity and Toxicity Neutralization Studies for the Asiatic Mountain Pit Vipers, *Ovophis convictus*, *Ovophis tonkinensis*, and Hime Habu, *Ovophis okinavensis*

Choo Hock Tan, Praneetha Palasuberniam and Kae Yi Tan

Table S1. Relative gel band intensity of *Ovophis* pit viper venoms.

Venom / Gel Section	*Ovophis convictus* (West Malaysia)	*Ovophis tonkinensis* (Northern Vietnam)	*Ovophis tonkinensis* (Southern China)	*Ovophis okinavensis* (Okinawa, Japan)
R1	20.41	31.77	27.20	21.84
R2	58.38	49.62	47.27	53.64
R3	21.21	18.61	25.53	24.52
